# Lost in the Forest, Stuck in the Trees: Dispositional Global/Local Bias Is Resistant to Exposure to High and Low Spatial Frequencies

**DOI:** 10.1371/journal.pone.0098625

**Published:** 2014-07-03

**Authors:** Gillian Dale, Karen M. Arnell

**Affiliations:** Department of Psychology, Brock University, Ontario, Canada; University of Groningen, Netherlands

## Abstract

Visual stimuli can be perceived at a broad, “global” level, or at a more focused, “local” level. While research has shown that many individuals demonstrate a preference for global information, there are large individual differences in the degree of global/local bias, such that some individuals show a large global bias, some show a large local bias, and others show no bias. The main purpose of the current study was to examine whether these dispositional differences in global/local bias could be altered through various manipulations of high/low spatial frequency. Through 5 experiments, we examined various measures of dispositional global/local bias and whether performance on these measures could be altered by manipulating previous exposure to high or low spatial frequency information (with high/low spatial frequency faces, gratings, and Navon letters). Ultimately, there was little evidence of change from pre-to-post manipulation on the dispositional measures, and dispositional global/local bias was highly reliable pre- to post-manipulation. The results provide evidence that individual differences in global/local bias or preference are relatively resistant to exposure to spatial frequency information, and suggest that the processing mechanisms underlying high/low spatial frequency use and global/local bias may be more independent than previously thought.

## Introduction

**Figure 1:**
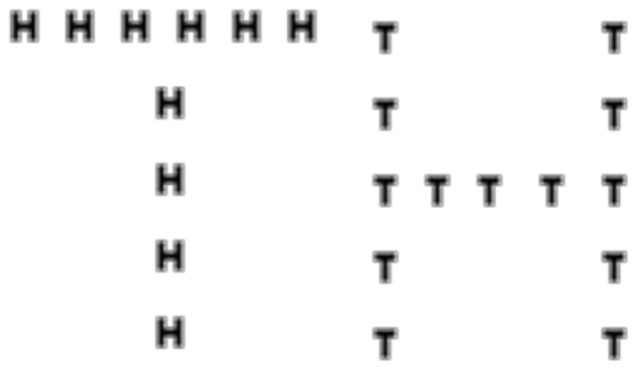
Traditional Navon letters.

Visual stimuli can be perceived at a broad, global level (e.g., “the forest”) or at a more focused, local level (e.g., “the trees”). This is referred to as “global/local processing”, and is commonly assessed through the use of compound stimuli [1]. The most frequently used global/local stimuli involve compound letters known as “Navon letters” [1], [2]. Navon letters are large, single letters (representing the global perceptual level) that are comprised of smaller letters (representing the local perceptual level) (see Figure 1). The global and local elements can either be congruent (e.g., a large “T” comprised of smaller “T's”), or incongruent (e.g., a large “T” comprised of small “S's”). A typical Navon task presents a single compound Navon letter on each trial, and requires the participant to identify either the large, global letter, or the small, local letters, as quickly as possible. The response time (RT) for detecting a given target letter appearing at the global versus local level is sometimes compared (e.g., [3]), but more often measures of global and local interference (i.e., the difference in RT from incongruent to congruent trials) are compared for local and global trials respectively. In addition to letter stimuli, variations in hierarchical stimuli have included shapes [4]–[6], digits [7], and objects [8].

Global/local processing can also be assessed through the use of forced-choice, non-speeded tasks in which the global and local levels are pitted against each other, and the participant determines which level is attended (e.g., [6]). For example, participants may be shown a square made of triangles and then asked to select either a triangle made of triangles, or a square made of squares, as the best representation of what they have just seen. Global or local preference is then assessed as the number of times a participant chooses the shape consistent with the global or local form. Interestingly, some of the early studies of global/local processing have suggested that, although stimuli can be viewed at either level, there is an overall global processing advantage [1], [2]. That is, the global information tends to be processed faster, earlier, and there is typically more interference from the global level when focusing on the local level, as compared to the reverse [1]. Additionally, when using a non-speeded forced-choice task, individuals as a whole are more likely to select or attend to the global figure, as compared to the local. This suggests that the processing of the coarse, global form of a stimulus takes precedence over the processing of the detailed, local parts [2]. This phenomenon is referred to as the “global advantage” or the “global precedence effect”, and suggests that visual processing occurs in a coarse-to-fine manner. However, this global advantage is neither universal nor absolute, and can be altered in a myriad of ways.

One of the most commonly known ways of altering the global advantage is by changing the stimulus or task parameters in such a way as to make the global form less salient. For example, changes to the overall visual angle [9], the aspect ratio of the local to global items [10], [11], or the exposure duration [12] can reduce, or even eliminate, the global advantage. Additionally, there is clear evidence for level-repetition effects, such that individuals are faster to respond to a globally- or locally-directed trial if they were directed to the same level on the preceding trial [13]–[18] (see also [19]). As such, global/local biases can clearly be manipulated or altered by simple task and stimulus changes.

### Influencing Global/Local Bias

The degree of global or local bias within an individual can not only be influenced by the stimulus or task parameters, but also by individual characteristics and behaviours that are related to a broadened or narrowed attentional focus. For example, studies have suggested that positive affect may have a broadening effect on attention [20] and negative affect a narrowing effect [21]. To examine the effect of negative affect on attentional breadth, Gasper and Clore [5] induced one of three mood states (sad, happy, or neutral) prior to participants completing a forced-choice global/local preference task. Participants induced into a sad mood state had lower global scores than did the participants in the happy or neutral groups, and were also more likely to report making their choices in this task based on the local information, rather than the global. Correspondingly, Fredrickson and Branigan [4] found that induced positive mood states resulted in larger global scores on the same global/local preference task as used in Gasper and Clore [5]. However, Gable and Harmon-Jones [3], [22] used a global/local task with compound stimuli and observed that, regardless of valence, induced affective states that were low in approach motivation (e.g., amusement, sadness) led to diffusion or broadening of attention, whereas induced states that were high in approach motivation (e.g., desire, disgust) led to a narrowing of attention (see [23] for a review), suggesting that it is motivational intensity that modulates global/local bias.

In addition to broadening and narrowing attention through affect, global/local bias has been modulated using other tasks designed to broaden attentional scope. For example, Liberman and Förster [24] showed in three studies that participants who had been primed to think of the distant future, distant spatial locations, and distant social relationships were faster at responding to global Navon letters than individuals who had either not been primed, or who had been primed to imagine proximal distances, locations, and relationships. Another recent study showed that when individuals are asked to perform a task (such as solving anagrams or navigating a maze), and they are presented with “obstacles” (such as distracting background noises framed as obstacles to be overcome, or physical obstacles in the maze), they tended to broaden their attentional scope in order to discover a new path around the obstacle, and later show faster responses to global stimuli [25].

### Global/Local Bias Influencing Performance on Face Perception

While much of the research on global/local processing has focused on how global/local biases can be altered, researchers have also focused on how an induced global or local state can influence performance on other tasks. For example, individuals who have been induced into a globally biased state using Navon stimuli are better at detecting sarcasm [26], detecting similarities between dissimilar television shows and objects [27], self-regulating [28], and at making basic and subordinate-level object discriminations from within similar distractors [29]. (see [30] for a more exhaustive list). However, the area in which global/local processing has been shown to play the most prominent role is in face recognition and perception.

Face recognition is thought to rely more on holistic processing than does the recognition of other objects. Specifically, faces are thought to be processed in a holistic manner that uses the entire face configuration, rather than individual features, during recognition [31]. This holistic processing can be disrupted in a variety of ways; most notably by inverting the face and thus disrupting the perception of the configural relationships [32]. Holistic processing is considered by many to be identical to global processing, such that both refer to the idea that an object (or face) is viewed in terms of its whole, rather than its constituent parts [1]. While there is currently debate over whether these terms can be used interchangeably (see [33]), many studies have sought to examine the effect of altering global/local processing biases on face recognition.

Macrae and Lewis [34] had participants watch a 30 sec. video of a simulated robbery, after which they viewed hierarchical Navon letters and reported the identity of the letter at either the global or local level for 10 minutes (control participants completed an unrelated filler task). After this induction task, participants viewed a lineup of 8 faces, which included the robber's face, and were asked to identify the robber from the initial video clip. Interestingly, the locally focused group showed impairments in face recognition, whereas the globally focused group showed enhancements in face recognition, relative to controls.

Perfect [35] later replicated this effect, but had half of the participants perform first a global, and then a local, task, whereas the other group performed a local task first, followed by a global task. Whichever global/local level the participants attended to last influenced their face identification accuracy, such that participants who performed the global task last had enhanced face identification accuracy, whereas those who performed the local task last showed diminished face identification accuracy (relative to controls). Similarly, Hills and Lewis [33] showed a reduction in face identification accuracy following the processing of the local elements of Navon letters, and also when biasing participants into a locally focused state using global/local shapes, such as diamonds and squares.

Weston and Perfect [36] showed that inducing participants into a globally or locally focused state using a Navon letter task can influence performance on the composite face task. In the composite face task participants are presented with faces that consist of the top half of one identity and the bottom half of another identity [36]. On some of the trials the face halves are aligned, and on some they are misaligned. Participants are generally slower to make old/new identifications for half of the face when the two face halves are aligned as compared to when they are misaligned. This *composite face effect* is thought to occur because intact faces are processed holistically and aligning the face halves of two identities creates the impression of a novel face, rather than two individual face parts.

In the Weston and Perfect [36] study, participants were given a global/local task in which they were either instructed to respond to only the local information (i.e., the local group), or the global information (i.e., the global group), and then complete a composite face task. Control participants performed a separate non-global/local task before the composite face task. Interestingly, the individuals who were in the local manipulation group were significantly faster at identifying whether a top or bottom face half was old or new on aligned trials as compared to both the global and control groups (who did not differ from each other), thus demonstrating that the local induction reduced the composite face effect. This suggests that a local processing style is useful for featural identification, whereas a global processing style is better for holistic identification (as with normal, intact faces). Gao, Flevaris, Robertson and Bentin [37] showed a similar effect with the composite face task using a trial-by-trial manipulation, such that participants reported either the local or global level of a Navon stimulus immediately before completing a face trial.

### Individual Differences in Global/Local Preference

The finding that global/local performance can be altered in a myriad of ways, and in turn can influence performance on other cognitive tasks, implies that global/local biases are dependent upon the tasks themselves, or the state of the participant during testing. However, individuals also vary naturally in their degree of global/local bias. For example, individuals from remote cultures [38] and musicians [39] tend to show a local bias, and individuals who follow a religion that emphasizes individualism (i.e., Calvinism) show a smaller global precedence effect than do atheists or Catholics [40]. Similarly, individuals with disorders such as obsessive-compulsive disorder [41] and autism [42] also tend to show larger local than global biases. There are also reported effects of age [43], and race [44] on global/local preference.

A recent study directly examined these individual differences in order to determine how stable dispositional global/local biases were over time [45]. Over two experiments, Dale and Arnell [45] showed the dispositional global/local biases, as assessed by a traditional Navon letter interference task, the Kimchi and Palmer [6] forced-choice shape task, and a forced-choice task with high/low spatial frequency hybrid faces [46], remained stable over a period of 7–10 days, suggesting that these individual differences are trait-like, and may reflect some default processing strategy. As such, it is clear that although global/local processing biases can be altered, or even manufactured, by altering stimulus and task parameters, individuals also show a large degree of variation person to person, and that this variation remains relatively stable over time. Additionally, these dispositional differences in global/local bias have been shown to relate to individual differences on other cognitive tasks, such as the attentional blink [47] which suggests that not only do individuals vary in their preference for global or local information, but that this variation influences performance on other cognitive tasks.

### Spatial Frequency and Global/Local Preference

It is clear that global/local processing biases can be altered by a variety of stimulus and task manipulations, as well as by manipulating the state of the participant. Additionally, global and local processing have been shown to influence performance on a variety of other non-global/local tasks, particularly tasks of face processing, presumably by biasing an individual's global/local disposition in the direction of the attended global or local level. Global/local processing has also been linked to spatial frequency, such that low spatial frequencies are linked to global processing and high spatial frequencies to local processing [17]. Specifically, global stimuli have been found to carry contain mainly low spatial frequency information, whereas local stimuli carry mainly high spatial frequency information [17], [48], [49]. Additionally, Shulman, Sullivan, Gish, and Sakoda [48] showed that adapting participants to low spatial frequencies aided the detection of global figures, whereas adapting participants to high spatial frequencies aided the detection of local figures (although participants as a whole were faster to respond to the global figures, regardless of the adaptation). This suggests that global/local and spatial frequency may be related to the same underlying mechanism, and that low and high spatial frequencies can facilitate perception of, and attention to, global and local forms respectively.

However, some researchers hypothesize that spatial frequency is related to, but distinct from, global/local processing, and that the spatial frequencies of the global/local stimuli, rather than the “globalness” or “localness” itself, might be contributing to effects such as those reported above [17]. For example, Hills and Lewis [50] showed that there was a large decrement in face recognition accuracy after the repeated presentation of local Navon trials, and that faces that were presented with only the high spatial frequency information intact were more difficult to identify, supporting the idea that a local processing bias reduces face recognition accuracy, whereas a global processing bias enhances face recognition accuracy. However, they also showed that when the Navon stimuli were blurred, or were of low contrast, this recognition deficit disappeared. Furthermore, they demonstrated that there is no recognition deficit for high-pass filtered faces if they are preceded by global Navon trials. This suggests that spatial frequency and global/local do not necessarily share the same underlying mechanism, and that adaptation to spatial frequencies and contrast, rather than “local-ness” or “global-ness”, lead to the differences in face recognition accuracy following the presentation of global/local Navon stimuli. In addition, Dale and Arnell [45] showed that while individual differences in tendency to use high or low spatial frequency information, and global/local bias in a hierarchical shape task were both highly reliable individual difference measures across 1 week, performance on these two tasks was unrelated. The tendency to use high or low spatial frequency information was also unrelated to global interference on the Navon letter task. This suggests again that the tendency to use low versus high spatial frequencies and the tendency to attend to the global versus local level do not come from a common source, and that global/local preference may not be influenced by spatial frequency in the manner that is often assumed.

### The Current Study

To our knowledge, no study has yet examined whether exposure to global/local or high/low spatial frequency stimuli can temporarily alter an individual's dispositional global/local response bias (i.e., can viewing low spatial frequency information make one temporarily more global, or can viewing high spatial frequency information make one more local?). As such, the central purpose of the current study was to examine, through five different experiments, whether individual differences in dispositional global/local bias can also be temporarily altered through exposing participants to high/low spatial frequency information, and Navon letters. If attending to high spatial frequencies can bias subsequent processing to be local, and/or attending to low spatial frequencies can bias subsequent processing to be global, then we will have found a simple and efficient way to modulate global/local bias. However, if separate mechanisms underlie the preference to use low versus high spatial frequency information and global/local preference, as suggested by Hills and Lewis [50], and Dale and Arnell [45], then exposure to specific spatial frequency information may be unable to alter global/local preference.

In order to manipulate spatial frequency in Experiments 1 and 2, we used two types of stimuli that, when attended, have previously been shown to influence performance on non-global/local tasks; presumably via changes in global/local processing bias. These stimuli are high/low spatial frequency faces (Experiments 1a and b) and high/low spatial frequency gratings (Experiments 1a and b). Dispositional biases were measured using a hierarchical shape task (Experiments 1a and 2a), and a Navon letter task (Experiments 1b and 2b) as in Dale and Arnell [49]. In addition, we used classic Navon stimuli (Experiment 3) to manipulate dispositional global/local bias and spatial frequency use, as measured by a hierarchical shape task and a dispositional high/low spatial frequency face task as in Dale and Arnell [49] (see Table 1 for a break-down).

**Table 1:** Manipulation tasks, dispositional tasks, and ANOVA results for each experiment.

Experiment	Manipulation	Dispositional	Main Effect Pre/Post	Main Effect SF	Interaction
1a	High/Low Faces	Paper Shape	*F*(1, 44) = 2.66	*F*(1, 44) = 0.26	*F*(1, 44) = 0.15
			*p* = .11	*p* = .61	*p* = .70
			η_ρ_^2^ = .06	η_ρ_^2^<.01	η_ρ_^2^<.01
1b	High/Low Faces	Navon Letters	*F*(1, 38) = 1.48	*F*(1, 38) = 0.18	*F*(1, 38) = 0.22
			*p* = .23	*p* = .67	*p* = .64
			η_ρ_^2^ = .04	η_ρ_^2^<.01	η_ρ_^2^<.01
2a	Hi/Low Gratings	Paper Shape	*F*(1, 43) = 0.43	*F*(1, 43) = 0.65	*F*(1, 43) = 0.30
			*p* = .52	*p* = .42	*p* = .58
			η_ρ_^2^ = .01	η_ρ_^2^ = .01	η_ρ_^2^ = .01
2b	Hi/Low Gratings	Navon Letters	*F*(1, 38) = 4.97	*F*(1, 38) = 0.60	*F*(1, 38) = 3.48
			*p* = .03	*p* = .44	*p* = .07
			η_ρ_^2^ = .12	η_ρ_^2^ = .02	η_ρ_^2^ = .08
3	Navon Letters	Paper Shape	*F*(1, 22) = 0.01	*F*(1, 22) = 0.01	*F*(1, 22) = 0.09
			*p* = .92	*p* = .97	*p* = .76
			η_ρ_^2^<.01	η_ρ_^2^<.01	η_ρ_^2^<.01
		Faces	*F*(1, 22) = 0.71	*F*(1, 22) = 0.09	*F*(1, 22) = 2.34
			*p* = .41	*p* = .77	*p* = .14
			η_ρ_^2^ = .03	η_ρ_^2^<.01	η_ρ_^2^ = .10

## Introduction: Experiment 1a and 1b

Experiment 1a and 1b were designed to examine whether exposure to high/low spatial frequency faces can alter dispositional global/local biases as measured by a hierarchical shape task (Experiment 1a) and a Navon letter task (Experiment 1b). Spatial frequency faces were chosen for the manipulation task because, unlike traditional global/local measures, the high/low SF faces allow us to present participants with either the global or the local level in isolation. This makes them a particularly appropriate tool for biasing perceptual breadth given that these stimuli do not expose participants to both global/local levels at once. Two different dispositional measures were used in order to examine pre/post manipulation biases, both for a well-established measure of global/local processing bias (i.e., Navon letters in Experiment 1b), as well as a highly reliable measure of global/local bias (i.e., hierarchical shapes in Experiment 1a). To examine the flexibility of dispositional global/local biases, participants were induced into both a global and a local state in two different blocks (counterbalanced). The dispositional measures were administered before and after the manipulation task in both blocks in order to examine post-manipulation differences in global/local bias.

## Methods: Experiment 1a and 1b

### Ethics Statement

In this, and all following experiments, participants provided written consent before participating. This research was approved by the Brock University Research Ethics Board (REB approval code 08-045).

### Participants

A total of 86 Brock University undergraduate student volunteers participated in Experiment 1 for extra course credit: 46 participants (5 males) for Experiment 1a and 40 participants (3 males) for Experiment 1b. The participants ranged in age from 18 to 26 years (*M* = 19.7, *SD* = 2.2). All of the participants in this experiment, and in the following experiments, reported normal or corrected-to-normal vision and having learned English before the age of 8. All experiments were conducted one-on-one with each participant, and took approximately one hour to complete.

### Apparatus

The computerized tasks in all of the experiments reported herein were presented using a Dell dual core desktop computer with a 17 inch CRT monitor, and were programmed and controlled using E-Prime software (Psychology Software Tools Inc.). The participants made responses via manual button-press on the computer keyboard.

### Stimuli and Design

#### Dispositional Task for Experiment 1a: Global/Local Shapes

**Figure 2:**
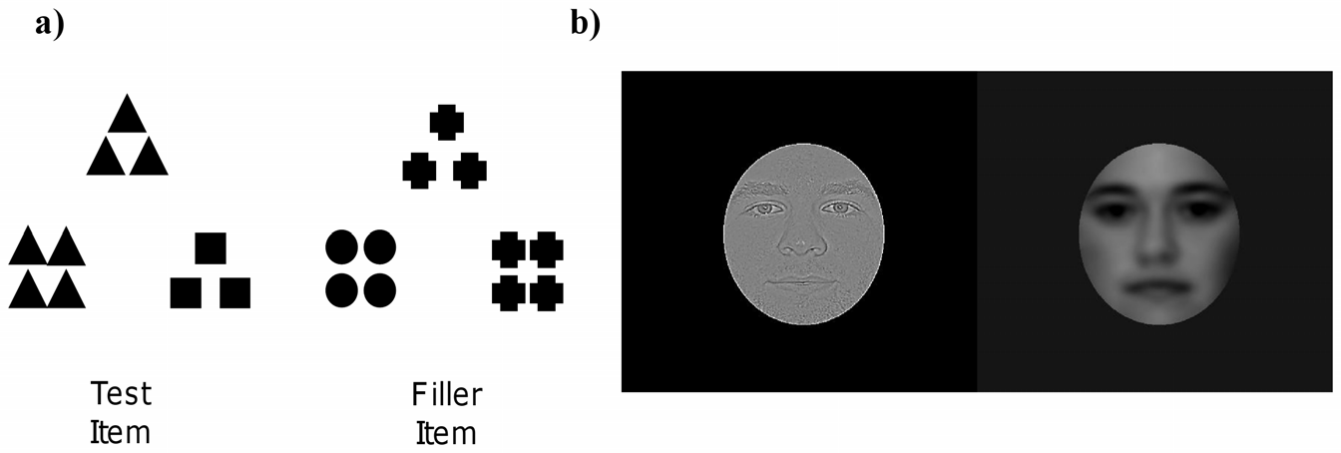
Sample stimuli from the dispositional and manipulation global/local tasks. (a) Sample stimuli from the dispositional global/local shape task. The test triad on the left contains both a global and a local option, whereas the filler triad on the right has only one possible answer, and was not used to measure global/local processing. (b) Sample stimuli from the global/local face manipulation task. A HSF (local) face on the left side and a LSF (global) face on the right.

Participants in Experiment 1a completed the global/local shape dispositional task, which was used to assess participants' degree of global or local bias, before and after each manipulation. For this paper-and-pencil task, participants were presented with a booklet that contained global/local shape triads adapted from Kimchi and Palmer [6] and Fredrickson and Branigan [4]. The shape triads were composed of three different hierarchical shapes that were arranged with a standard shape on top, and two comparison shapes on the bottom (see Figure 2a). The participants were instructed to quickly circle the comparison shape that they felt best matched the standard shape for each of the triads. The participants were asked to complete this task as quickly as possible, and were told to use their first instinct when selecting the comparison shape.

The task contained 8 test triads and 16 filler triads that were intermixed, for a total of 24 triads. The hierarchical shapes in each test triad consisted of 3–4 small (5×5 mm) square or triangle shapes (local level) that formed a larger (15×15 mm) square or triangle (global level). For the test triads both comparison figures matched the standard figure, but one matched at the global level (i.e., the overall shape outline matched the standard), and one matched at the local level (i.e., the smaller, detailed shape matched the standard), counterbalanced for presentation location. The hierarchical shapes in each filler triad were comprised of 3–4 small (5×5 mm) circles, squares, triangles, or crosses (local level) that formed a larger (15×15 mm) square or triangle (global level). For the filler triads, one of the comparison figures matched the standard shape at the global or local level, and the other did not match either level of the standard (location counterbalanced).

In order to obtain an index of global bias, the total number of test triads in which the global comparison shape was selected was calculated for each participant. This yielded a global score out of 8, where scores above 4 indicated a global bias, a score of 4 indicated a lack of preference for either the global or the local level, and scores below 4 indicated a local bias. Filler triads had only one correct response; therefore they were not used as an index of global/local bias.

#### Dispositional Task for Experiment 1b: Navon Letter Task

Participants in Experiment 1b completed a Navon letter dispositional measure of global/local bias before and after each manipulation. Navon stimuli were created in Adobe Photoshop, and consisted of large, global letters constructed of smaller letters (e.g., an H made out of T's) (see Figure 1). The global letters (60×45 mm) were 10 times as large as the smaller local letters (6×4.5 mm), and it took roughly 10 local letters to make up a single global letter. A total of four different Navon letters were created, half of which were congruent (i.e., global T's made of local T's and global H's made of local H's), and half of which were incongruent (global T's made of local H's and global H's made of local T's). All of the letters were presented in black New Courier font on a white background, and the viewing distance was approximately 55 cm unrestrained.

Each trial began with a 500 ms central fixation cross, after which a single Navon stimulus was presented in the center of the computer screen for 15 ms. After the letter was presented, a blank response screen was displayed. Global and local trials were presented in alternating blocks, with 24 trials in each of 4 blocks for a total of 96 trials. Participants were required to quickly report either the identity of the smaller letters (local trials) or the identity of the large letter (global trials) by pressing the corresponding key on the keyboard. Participants were urged to respond as quickly and as accurately as possible. Response time (RT) was recorded. The letter combinations were randomly presented within each block, and each letter was presented 6 times within each block. All participants began with the global block.

RTs for incorrect trials and RTs that fell outside three standard deviations from the mean per condition per participant were removed. Global interference scores were then calculated for each participant by examining the degree to which global features on the local incongruent trials interfered with RT (local incongruent RT – local congruent RT). High, positive global interference scores suggest a global processing bias, whereas low or negative global interference scores suggest either no global bias, or a local processing bias.

#### Manipulation Task: High/Low Spatial Frequency Faces

Whereas the participants from Experiments 1a and 1b completed different dispositional global/local tasks, both groups of participants completed the same manipulation task. In this task, participants were presented with high spatial frequency (HSF) and low spatial frequency (LSF) filtered faces. Twenty-one male and 21 female normed young adult faces with neutral expressions were obtained from The Center for Vital Longevity Face Database [51], and all of the models signed release forms consenting to the use of their image in publication The faces were cropped, converted to grayscale, and were pasted onto a 480×480 pixel dark grey background so that they subtended approximately 16° of visual angle with an unrestrained viewing distance of approximately 55 cm. A 215×275 pixel dark grey frame occluder was placed over each face to obscure the hair and ears. High and low spatial frequency faces were then constructed in Adobe Photoshop using these faces. High spatial frequency faces were constructed by using a high-pass filter in Photoshop, and contained only spatial frequencies higher than 6 cycles/degree of visual angle (i.e. a radius of 1.5 pixels). Low spatial frequency faces were constructed by using a Gaussian blur in Photoshop, and contained only spatial frequencies lower than 2 cycles/degree of visual angle (i.e. a radius of 4.5 pixels) (see Figure 2b). As each face was made into both a high and a low spatial frequency face, a total of 42 HSF and 42 LSF faces were created.

Each trial began with a 500 ms blank grey screen, after which either a high or a low spatial frequency face (depending on the experimental block) appeared in the center of the screen. Participants were asked to indicate whether the face was male or female by pressing the corresponding key on the keyboard (“F” for female; “H” for male). The faces remained on the screen until the participant made a response, and participants were encouraged to respond as quickly as possible. Each participant performed a block of 496 high spatial frequency trials presented in random order, and a separate block of 496 low spatial frequency trials presented in random order, and each block took approximately 15 minutes to complete. An equal number of male and female faces were shown for each spatial frequency block. Accuracy was recorded to ensure that participants were performing the task appropriately.

### Procedure

Both Experiment 1a and 1b consisted of two experimental blocks: a high spatial frequency (local) manipulation block and a low spatial frequency (global) manipulation block, the order of which was counterbalanced across participants. Each block began with the administration of the either the global/local shape task (Experiment 1a) or the Navon letter task (Experiment 1b) in order to obtain a pre-manipulation measure of each participant's dispositional global/local bias. Participants from both experiments then completed the faces manipulation task with either the high or low spatial frequency stimuli. After completion of the first manipulation task, participants completed a second version of the dispositional global/local task, in order for us to examine any post-manipulation changes in global/local bias. Participants were then required to take a 5-minute break, during which they completed a maze task which was designed to reduce carryover effects from one block to the next [52]. After the break, they completed the second experimental block which, like the first block, included a pre and post-test dispositional global/local task, and a manipulation task with the opposite spatial frequency to that used in the first block.

### Analyses

For Experiments 1ab and 2ab, data for each of the dispositional tasks were first analyzed using a mixed-model ANOVA where high/low spatial frequency block and pre/post manipulation were within participants factors, and block order was a between participant factor. In all cases, there were no main effects or interactions with block order, and the data were collapsed across this factor. In order to examine whether repeated exposure to high and low spatial frequency faces or gratings could influence dispositional global/local bias scores, these scores were entered into a 2×2 repeated measures ANOVA with high/low spatial frequency and pre/post manipulation as factors. Note that successful biasing of dispositional global/local bias scores in the direction of the manipulation would result in an interaction where post-manipulation global scores would become more global after the low spatial frequency exposure, and less global after the high spatial frequency exposure. The results of these ANOVAs are summarized in Table 1 for each experiment.

## Results: Experiment 1a and 1b

### Experiment 1a

For the face manipulation task, mean gender discrimination accuracy was .80 (*SD* = .06) for the high spatial frequency condition and .85 (*SD* = .07) for the low spatial frequency condition, indicating that participants were performing the task as instructed.

**Figure 3:**
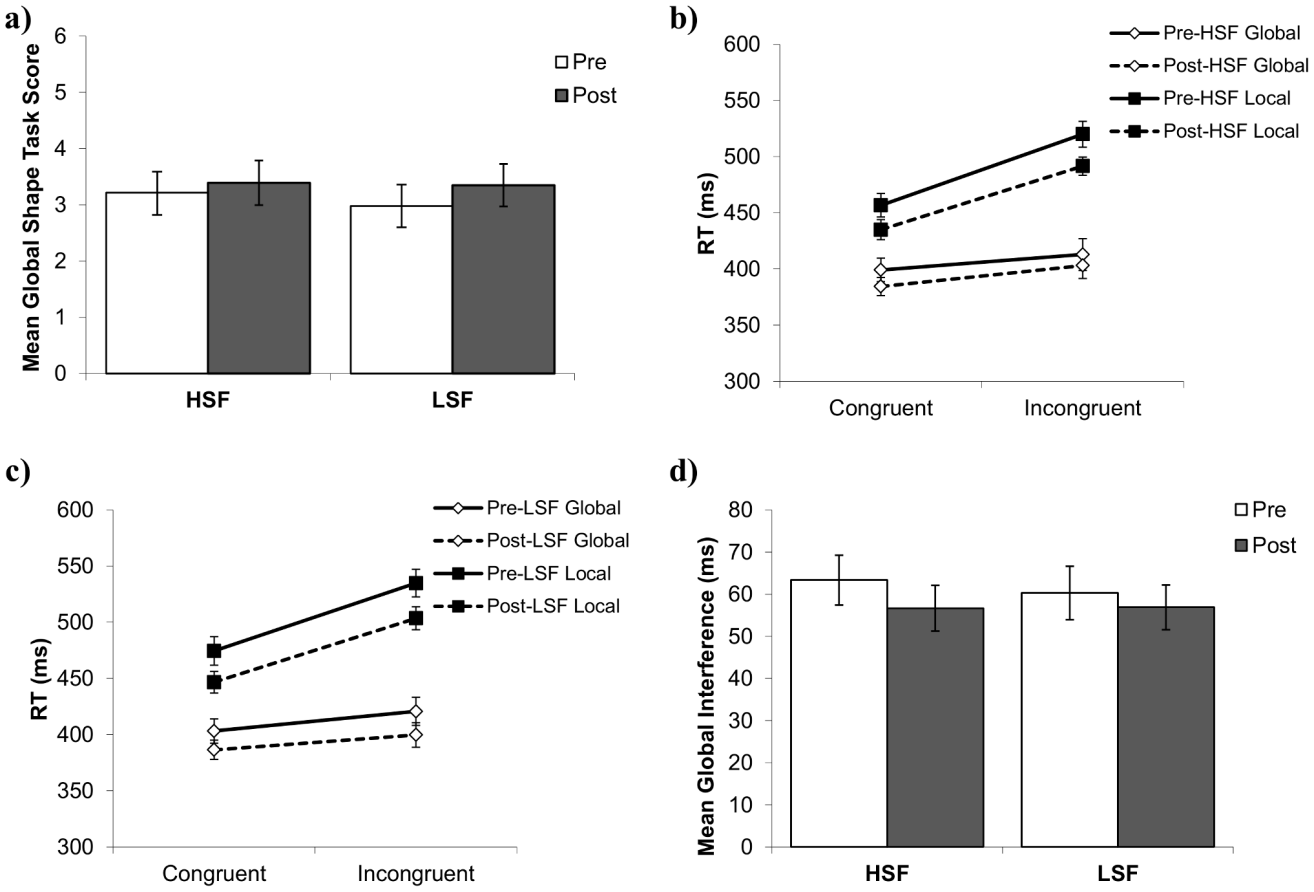
Experiment 1a and b pattern of means. (a) Experiment 1a mean pre- and post-manipulation global shape task scores as a function of manipulation frequency (HSF or LSF faces). (b) Experiment 1b mean RTs on the Navon letter task, as a function of pre- and post-manipulation, stimulus level (global or local), and target congruency in the HSF condition and (c) in the LSF manipulation condition. (d) Experiment 1b mean pre-and post-manipulation global Navon interference scores as a function of viewing LSF or HSF faces in the manipulation task. Error bars in this and all other figures represent the standard error for each condition mean.

The mean global scores on the hierarchical shape task are shown in Figure 3a as a function of high/low spatial frequency and pre/post manipulation. A repeated measures ANOVA showed that there was no main effect of pre/post or manipulation frequency, and no significant interaction between frequency and pre/post, indicating that the global shape scores were not influenced by the manipulation tasks (see Table 1). Indeed, planned comparisons showed no significant pre-to-post change in global shape scores when using high or low spatial frequency faces as a manipulation, *t*(45) = −0.84, *p* = .41, *d* = −.06 and *t*(45) = −1.31, *p* = .20, *d* = −.14 respectively.

### Experiment 1b

For the face manipulation task, mean gender discrimination accuracy was .86 (*SD* = .07) for the high spatial frequency condition and .88 (*SD* = .06) for the low spatial frequency condition, indicating that participants were performing the task as instructed.

The means for the Navon letter task are presented in Figure 3bc. When 2 (congruency) X 2 (attend global/local) ANOVAs were performed for each combination of pre/post and spatial frequency manipulation, all main effects and interactions were significant (all *p*'s<.001), demonstrating the expected global precedence effect in each administration of the task.

A 2 (high/low spatial frequency) X 2 (pre/post) ANOVA was performed on the global interference scores (see Figure 3d). The results showed no significant main effect of pre/post or manipulation frequency, and no interaction between these two variables (see Table 1). Additionally, planned comparisons showed no significant difference between pre- and post-manipulation scores for either the high spatial frequency block, *t*(39) = .87, *p* = .39, *d* = .19, or for the low spatial frequency block, *t*(39) = .54, *p* = .59, *d* = .09. Importantly, if we examine local, rather than global, interference, we find no significant main effect of either manipulation frequency or pre/post (all *p*'s>.91), and no interaction between frequency and pre/post (*p* = .47). Additionally, if we instead examine overall global and local RT, rather than the interference measure used here, we find no significant interactions among manipulation frequency, stimulus level (global/local), and pre/post manipulation RT (all *p*'*s*>.10). Therefore, these findings are not due to using an interference measure, rather than an overall RT measure.

### Pre/Post Correlations

Importantly, the global scores on the hierarchical shape task were not simply random, but appeared to be reliable measures of an individual's global bias over time. Indeed, Experiment 1a pre- and post-manipulation global shape scores correlated .86 and .72 for the high and low spatial frequency manipulation conditions respectively, indicating that the global score on the hierarchical shape task is a reliable measure of dispositional global/local bias and that individual differences are stable within a single test session. The Navon letter task scores in Experiment 1b, however, were less reliable such that pre-and post-manipulation global interference scores for the low spatial frequency condition were significantly correlated at .44, whereas the correlation between pre/post interference scores for the high spatial frequency condition was not significant (*r* = .09).

## Discussion: Experiment 1a and 1b

In this experiment, we were unable to effectively manipulate individuals' global/local processing, as measured by the global shape task (Experiment 1a), and the Navon letter task (Experiment 1b), by exposing participants to high/low spatial frequency faces. This suggests that dispositional global/local biases may be resistant to very recent exposure to spatial frequency information. However, it is possible that the face manipulation task itself was not appropriate for evoking change in attentional breadth. Although participants were required to view high and low spatial frequency faces, they were not necessarily required to focus on the frequency information itself in order to make a face-gender judgment. As such, this may have prevented the participants from being adapted to the high and low spatial frequency during this task. Therefore, we conducted Experiment 2a and b in which participants were presented with a more “pure” spatial frequency task where they were required to view high/low spatial frequency gratings. In this task, participants are required to direct their attention to the gratings themselves, and make judgments about the orientation of the lines within the gratings. This requires the participants to use, and adapt to, the spatial frequency for each condition in order to actually perform the task.

## Methods: Experiment 2a and 2b

### Participants

A total of 84 Brock University undergraduate student volunteers participated in Experiment 2 for extra course credit: 45 participants (11 males) for Experiment 2a, and 39 participants (5 males) for Experiment 2b. The participants ranged in age from 18 to 25 years (*M* = 19.3, *SD* = 1.75).

### Stimuli and Design

The participants in Experiment 2a completed the same dispositional task as in Experiment 1a (i.e., the global/local shape task), whereas the participants in Experiment 2b completed the same dispositional task as in Experiment 1b (i.e., the Navon letter task). For the manipulation task, however, participants from both Experiment 2a and 2b completed a high/low spatial frequency grating task.

#### Manipulation Task: High/Low Spatial Frequency Gratings

**Figure 4:**
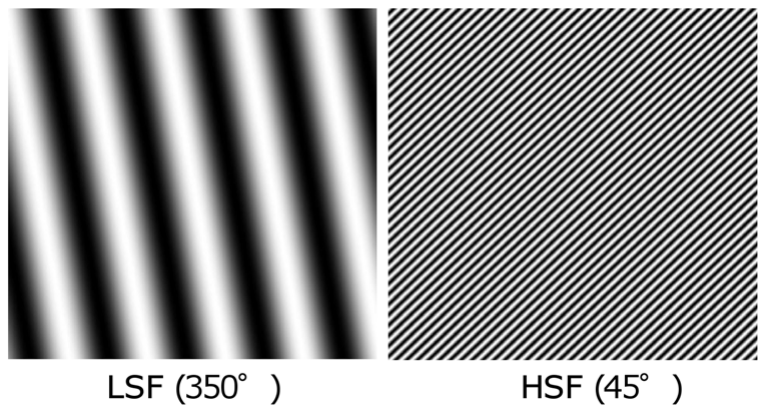
Sample stimuli from the SF grating manipulation task. The grating on the left is a LSF (global) grating with a 350° tilt, whereas the one on the right is a HSF (local) grating with a 45° tilt.

Participants in Experiment 2a and 2b completed a manipulation task in which they were presented with high and low spatial frequency gratings. The gratings were created using online software developed by Sebastiaan Mathôt [53]. All of the grating stimuli were 480×480 pixels in size, were presented at 100% contrast, and subtended approximately 6.6° of visual angle with an unrestrained viewing distance of approximately 55 cm. The gratings were either 7.2 cycles/degree (10 pixels/cycle) for the high spatial frequency gratings, or .76 cycles/degree (1 pixel/cycle) for the low spatial frequency gratings. The gratings were tilted in 1 of 6 orientations: 10° (slight right), 45° (moderate right), 80° (extreme right), 280° (extreme left), 315° (moderate left), or 350° (slight left) (see Figure 4). Therefore, there were 6 high spatial frequency and 6 low spatial frequency gratings generated for a total of 12 gratings.

Each trial began with a 500 ms blank gray screen, after which either a high or low spatial frequency grating (depending on the experimental block) appeared in the center of the screen. Participants were required to indicate the direction in which the bars were leaning by pressing one of 6 labeled keys on the keyboard (3 =  extreme left, 4 =  moderate left, 5 =  slight left, 7 =  slight right, 8 =  moderate right, and 9 =  extreme right). The gratings remained on the screen until the participant made a response, and the participants were encouraged to respond as quickly and as accurately as possible. The high and low spatial frequency grating blocks each contained 300 trials presented in random order, and took approximately 10 minutes to complete. Accuracy on this task was measured to ensure that participants were performing the task as instructed.

### Procedure

The procedure for Experiment 2 was the same as in Experiment 1, with the exception that participants now completed the high/low spatial frequency grating manipulation task, rather than the face manipulation task used in Experiment 1ab.

## Results: Experiment 2a and 2b

### Experiment 2a

Mean orientation discrimination accuracy for the high spatial frequency grating manipulation task was .77 (*SD* = .23), and the mean accuracy for the low spatial frequency grating manipulation task was .81 (*SD* = .22), indicating that participants were attending to the gratings during the manipulation task.

**Figure 5:**
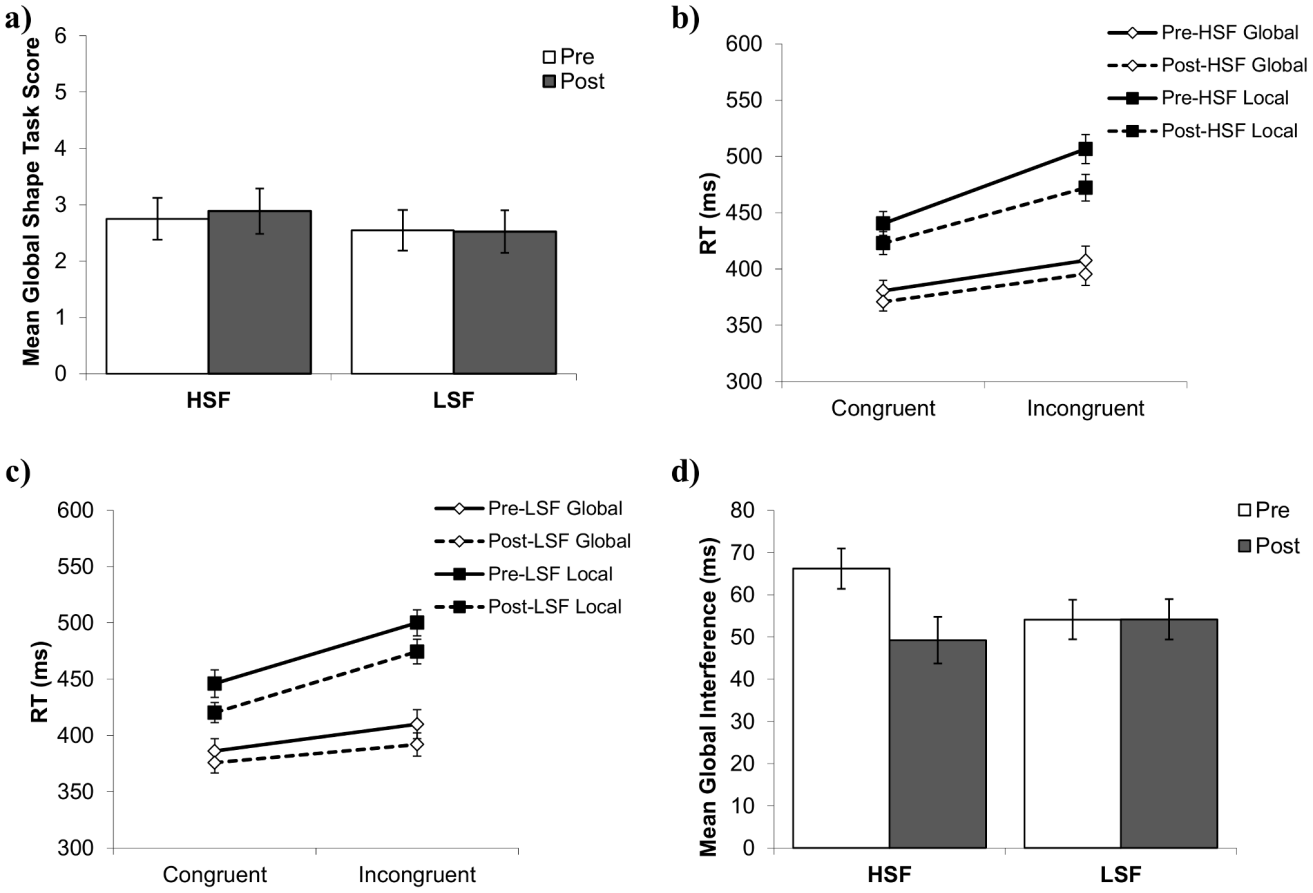
Experiment 2a and b pattern of means. (a) Experiment 2a mean pre- and post-manipulation global shape task scores as a function of viewing LSF or HSF gratings in the manipulation task. (b) Experiment 2b mean RTs on the Navon task, as a function of pre- and post-manipulation, stimulus level (global or local), and target congruency in the HSF condition and (c) in the LSF manipulation condition. (d) Experiment 2b mean pre-and post-manipulation global Navon interference scores as a function of viewing LSF or HSF gratings in the manipulation task.

The mean global shape scores are presented in Figure 5a as a function of high/low spatial frequency and pre/post manipulation. As with Experiment 1a, a repeated measures ANOVA showed no main effect of manipulation frequency or pre/post, and no significant interaction between these variables (see Table 1). Planned comparisons showed no significant difference in global shape scores from pre- to post-manipulation when using high or low spatial frequency gratings as a manipulation, *t*(43) = −0.62, *p* = .54, *d* = −.06, and *t*(43) = 0, *p* = 1.0, *d* = 0 respectively.

### Experiment 2b

Mean orientation discrimination accuracy for the high spatial frequency grating manipulation task was .85 (*SD* = .13), and the mean accuracy for the low spatial frequency grating manipulation task was .88 (*SD* = .09), indicating that participants were attending to the gratings during the manipulation task.

The means for the Navon task are presented in Figure 5b and c. When 2 (congruency) X 2 (attend global/local) ANOVAs were performed for each combination of pre/post and spatial frequency manipulation, all main effects and interactions were significant (all *p*'s<.004), demonstrating the expected global precedence effect in each administration of the task.

A 2 (HSF/LSF) X 2 (pre/post) ANOVA was performed on the global interference scores (see Figure 5d). The results again showed no significant main effect of manipulation frequency, but there was a significant main effect of pre/post, such that post-manipulation interference scores were lower than pre-manipulation interference scores. In addition, the interaction approached significance and the means showed the predicted pattern of effects (see Table 1). Finally, planned comparison showed no significant difference between pre- and post-manipulation scores in the low spatial frequency block, *t*(38) = −.01, *p* = .99, *d* = −.002, but there was a significant difference between the pre- and post-manipulation scores in the high spatial frequency block, *t*(38) = 2.92, *p* = .006, *d* = .50. As with Experiment 1, if we instead examine local, rather than global, interference, we find no significant main effect of either manipulation frequency or pre/post (all *p*'s>.37), and no interaction between frequency and pre/post (*p* = .54). However, if raw global and local RT scores are instead used, there was a significant interaction between stimulus level and pre/post (*p* = .03), but importantly there were no significant interactions between manipulation frequency and pre/post, frequency and stimulus level, or a three-way interaction (all *p*'s>.52). Therefore, these findings are not due to using an interference measure, rather than an overall RT measure.

Measures of global precedence (i.e., the difference between global and local interference) were also obtained and examined. In both Experiment 1b and 2b, the same pattern of results was found whether using global interference scores or global precedence scores. However, as global precedence is calculated as the difference score of two difference scores, and is thus less reliable, it is not as clear whether the inability to manipulate these scores is because they are stable, or if it is simply because there is too much measurement error. As such, these findings are not presented here.

### Pre/Post Correlations

Finally, we examined the correlation between the pre-and post-manipulation scores for each of the dispositional tasks, for each manipulation condition. The mean correlation between the pre-and post-manipulation global shape scores in Experiment 2a was .72 for the high spatial frequency condition, and .86 for the low spatial frequency condition. This shows a high degree of correspondence between the global shape scores before and after each manipulation, indicating that this task is a reliable measure of global bias. In Experiment 2b, while the global interference scores were moderately and significantly correlated for the HSF condition (*r* = .35), they were not significantly correlated for the LSF condition (*r* = .23). These correlations for both tasks approximate the reliability scores found by Dale & Arnell [45] for these tasks across 1 week, suggesting that the pre/post correlations were not inflated by participants remembering the responses they gave in the pretest and using them again in the post-test.

## Discussion: Experiment 2a and 2b

As with Experiment 1, we were again unable to successfully alter global/local biases, as measured by the global shape task (Experiment 2a) and the Navon letter task (Experiment 2b), by exposing individuals to high/low SF gratings. Therefore, it can be concluded that, when assessed by a dispositional shape task or Navon letters, dispositional global/local bias is resistant to exposure to very recent spatial frequency information, at least when using high/low spatial frequency faces and gratings as manipulation tools.

We did, however, find a significant difference in the pre-post-manipulation global interference scores for the high spatial frequency manipulation condition in Experiment 2b, accompanied by the numerically opposite pattern for the low spatial frequency condition, although with no significant interaction. One possible explanation for why this pre/post difference was found in only this experiment is that there may be more potential to show a change in dispositional global/local processing with the Navon letter task than with the shape task, given that global interference on the Navon letter task is less of a reliable trait variable than global scores in the shape task. Indeed, this speculation would fit with the results of Dale and Arnell [45] who showed high correlations in individual global shape scores across more than a week (*r* = .80), but lower, albeit significant, correlations for the Navon letter task across more than a week (*r*'s of .27 to .31). Additionally, when we examine the differences in the magnitude of global versus local interference in the Navon task, we find that there is an overall global precedence effect, such that participants were more biased toward the global than the local information (*p*<.004). As such, one possible explanation the finding that the HSF manipulation was more easily able to alter dispositional biases as compared to the LSF manipulation, because people were already quite global to begin with, and had less room to become even more global after the low spatial frequency manipulation. However, this explanation suggests that Navon scores should also have been influenced by exposure to high/low spatial frequencies in the HSF condition of Experiment 1b, but the interaction between pre/post and manipulation frequency did not approach significance, nor did the difference in pre-post global interference on the Navon task.

It is clear from these findings that dispositional global/local biases are difficult to alter when using high/low spatial frequency stimuli as a manipulation tool. One potential limitation of the above findings, however, is that the global/local manipulations were all completed within-subjects, such that we attempted to bias participants into both a global *and* a local state. Despite finding no consistent effects of block order, it is possible that carryover effects from the within design somewhat limited our ability to alter dispositional global/local bias. As such, we conducted a third experiment using a between-subjects design, such that some participants were biased with local stimuli and some with global.

Additionally, while the main purpose of this study was to determine whether exposure to high/low spatial frequency information could alter dispositional biases, many previous studies (e.g., [27], [34]–[36]) have used traditional Navon letters to bias participants, rather than the face or grating tasks previously employed in our experiments. As such, it is possible that our inability to influence dispositional global/local biases is the result of using SF manipulations, rather than being due to the fact that global/local biases are resistant to change by any global/local measure. Indeed, while SF appears to be related to global/local processing, and may facilitate global/local processing, there is evidence to suggest that they do not share the same underlying mechanism [45], [50]. Therefore, we used traditional Navon letter stimuli, rather than faces or gratings, as our manipulation task for Experiment 3. Finally, we included the high/low spatial frequency face task previously used in Dale and Arnell [45], adapted from Deruelle et al. [46] as a dispositional measure of low/high spatial frequency bias, allowing us to examine whether attending to global or local levels could influence subsequent spatial frequency use.

## Methods: Experiment 3

### Participants

Twenty-four (2 male) Brock University student volunteers participated in this experiment for extra course credit. Participants were randomly assigned to either the global manipulation group (N = 12) or the local manipulation group (N = 12). Participants ranged in age from 18 to 22 years (*M* = 19.9, *SD* = 1.2).

### Stimuli and Design

#### Dispositional Tasks: Shapes and Faces

All participants completed the same dispositional shape task used in Experiment 1a and 2a. In addition, participants completed a second dispositional task that used high/low spatial frequency face stimuli.

The high and low spatial frequency faces used in the Experiment 1 manipulation task were used to create high/low hybrid faces for the dispositional face task. These hybrid faces were created by taking the high spatial frequency version of one face and superimposing it over the low spatial frequency version of another face (matched for gender). Each face contributed high spatial frequency information to one hybrid face, and low spatial frequency information to another hybrid face, thus a total of 42 hybrid faces were constructed.

Each trial began with a 1000 ms blank screen, after which a hybrid face appeared for 300 ms in the center of the screen. The hybrid face was then replaced with the two original (unfiltered) faces that had comprised the hybrid face (i.e., the face that contributed the high spatial frequency information, and the face that contributed the low spatial frequency information). One of the unfiltered faces was presented on the left side of the screen, and one on the right (counterbalanced). Participants were instructed to indicate which unfiltered face best matched the hybrid face by pressing the corresponding key on the keyboard. The unfiltered faces remained on the screen until the participant made a response, and participants were encouraged to go with their first instinct and to not over-think their response. There were a total of 42 trials presented in random order, and the task took approximately 5 minutes to complete.

In order to calculate dispositional global/local bias, we totaled the number of trials out of 42 in which the participant chose the unfiltered face that had contributed low spatial frequency (global) information to the hybrid. This total represented an index of each participant's dispositional global bias, such that high global face scores indicated a global processing bias, and low global face scores indicated a local processing bias.

#### Manipulation Task: Global or Local Navon Letters

The Navon letter manipulation task was adapted from the traditional Navon interference task described in Experiment 1b and 2b. Navon stimuli were again created in Adobe Photoshop, but they were created differently depending on whether they were to be used in the global or local manipulation condition. For the global manipulation condition, the global letters (35×25 mm) were 10 times as large as the local letters (3.5×2.5 mm), and it took roughly 20 local letters to make up a single global letter. This resulted in dense, small Navon letters that were globally salient. Conversely, the letters for the local manipulation condition consisted of global letters (65×45 mm) that were 10 times as large as the local letters (6.5×4.5 mm). Approximately 9–12 local letters were used to make up each global letter, resulting in very sparse, large stimuli that were locally salient. A total of six different Navon letters (made of H's, T's, and F's) were created for each manipulation task, all of which were incongruent. All of the letters were presented in black New Courier font on a white background, and the viewing distance was approximately 55 cm unrestrained.

For the global manipulation task, each trial began with a 1000 ms fixation cross, after which a single Navon letter appeared in the center of the screen for 15 ms. The letter then disappeared and was replaced with a blank response screen. Participants were asked to indicate what the large, global letter was as quickly as possible by pressing the corresponding key on the keyboard (“H”, “T”, or “F”). The letters were presented in a random order, and each letter was presented 80 times for a total of 480 trials. RT and accuracy were recorded to ensure that participants were completing the task appropriately and were following directions.

The local Navon manipulation task was very similar, with the following exceptions. First, the stimuli were presented on the screen for 175 ms, rather than 15 ms, in order to give the participants the chance to better view the local letters. Second, participants in this group were asked to indicate what the small, local letters were, rather than the global letters.

### Procedure

At the beginning of the experiment, all participants completed the shape and face dispositional tasks, in order to provide us with an estimate of their pre-manipulation dispositional bias. They then completed one 480 trial block of either the global or the local manipulation task (depending on the group to which they had been assigned). After the first manipulation block, all participants completed a post-manipulation dispositional shape task. Next, participants completed a second 480-trial block of either the global or local manipulation task (reporting the same level as in the first manipulation block). Finally, participants completed a post-test dispositional face task.

## Results: Experiment 3

For the local manipulation group, mean accuracy for the first manipulation block was .96 (*SD* = .04) and accuracy for the second manipulation block was .96 (*SD* = .04). For the global manipulation group, mean accuracy for the first manipulation block was .96 (*SD* = .04) and accuracy for the second manipulation block was .96 (*SD* = .03).Therefore, the participants were performing the manipulation task as instructed, and with little difficulty.

**Figure 6:**
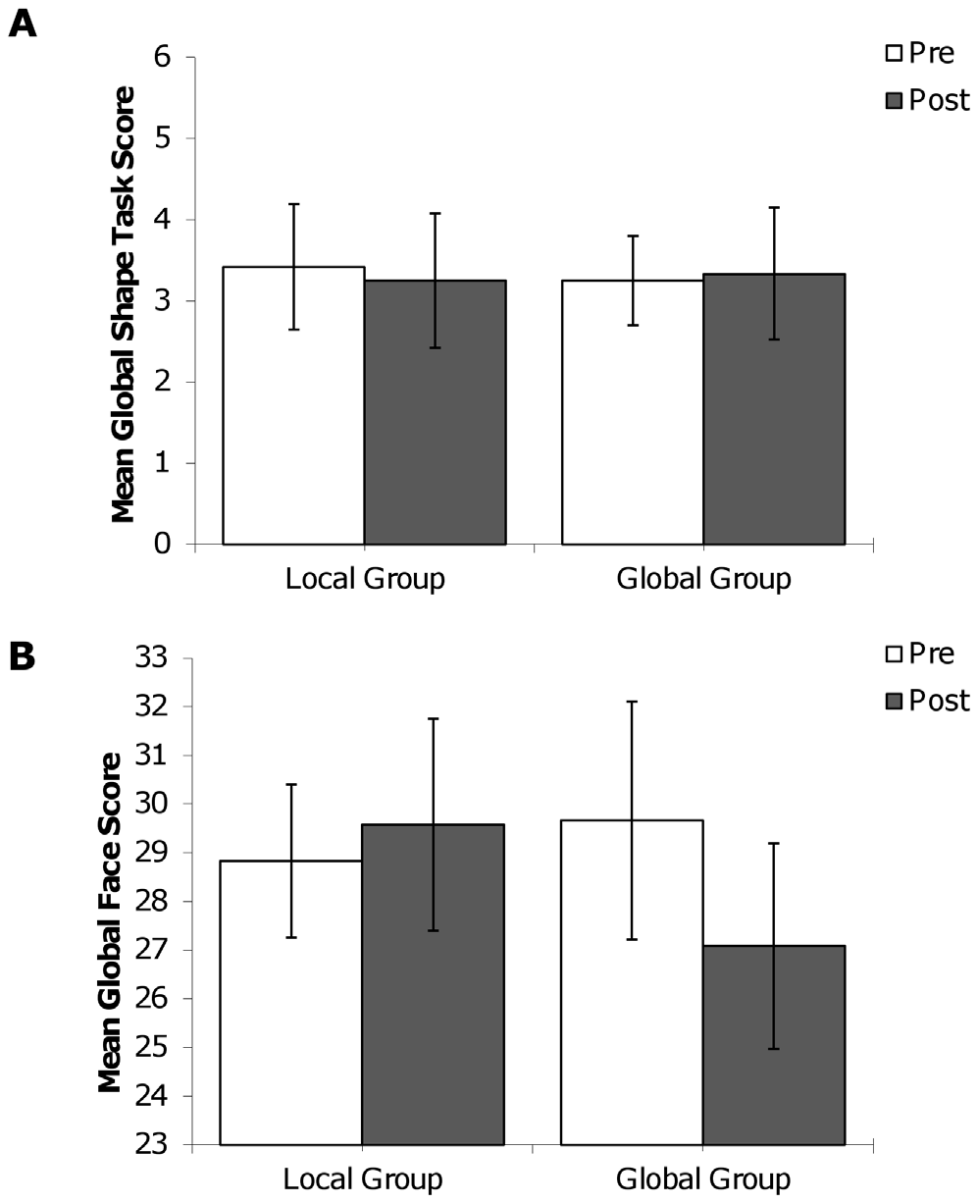
Experiment 3 pattern of means. (a) Experiment 3 mean pre- and post-manipulation global shape task scores as a function of Navon task manipulation group (global or local). (b) Experiment 3 mean pre- and post-manipulation global face scores as a function of Navon task manipulation group (global or local).

The means from the dispositional tasks are presented in Figure 6a (global shape scores) and Figure 6b (global face scores) as a function of pre/post and the assigned Navon level during the manipulation task. For each of the dispositional tasks, a 2×2 mixed model ANOVA was conducted with pre/post manipulation task as a within participants factor, and local/global level as a between participants variable. For the global shape task, results showed no significant main effect of either pre/post manipulation or local/global level, and no interaction between pre/post and local/global (see Table 1). Planned comparisons using paired-samples t-tests showed no significant difference between pre- and post-manipulation global shape scores for the local level, *t*(11) = .46, *p* = .66, *d* = .06, or the global level, *t*(11) = .11, *p* = .91, *d* = −.03.

For the face task, the ANOVA results again showed no significant main effect for pre/post manipulation or local/global level, and no interaction (see Table 1). Indeed, although the p-value for the interaction approached significance (*p* = .14), the pattern of means was the opposite of the predicted direction. There was no significant difference between pre- and post-manipulation face scores for the local level, *t*(11) = .53, *p* = .61, *d* = −.09, or the global level, *t*(11) = 1.57, *p* = .15, *d* = .39. Overall, it is clear that the manipulation task did not significantly alter dispositional global bias as measured by the shape or face tasks.

Finally, the correlations between pre- and post-manipulation scores on the shape and face tasks were examined. For the local group, pre- and post-manipulation shape task scores were correlated .90, whereas pre and post face task scores correlated .81. The correlations between pre- and post-manipulation scores were smaller in the global group, such that the shape task scores were correlated .47, and the face task scores correlated .66.

## Discussion: Experiment 3

The purpose of Experiment 3 was to rule out the possibility that the findings from the previous two experiments were simply due to the within-subjects design that we had employed. Additionally, we were also interested in seeing if the use of traditional Navon letters could affect change in global/local bias, as the previous manipulation tasks (faces and gratings) presented the participant with high/low SF information, rather than hierarchical stimuli. However, we were still unable to find a change from pre- to post-manipulation in either the global or the local manipulation group in this experiment. We have now shown the same pattern of null results using hierarchical shapes/faces/Navon letter tasks as dispositional global/local bias measures, and when using faces/gratings/Navon letters as manipulation tasks. This shows that our non-significant results are unlikely to be due to the type of dispositional measure, or the type of manipulation. This again suggests that dispositional global/local biases are stable and resistant to change, and that the null findings were not simply due to using spatial frequency manipulations.

## General Discussion

It is well documented that global/local performance can be altered through the use of task or stimulus manipulations (e.g., [9], [10], [12]), or through the use of an external, non-global/local task (e.g., [3]–[5], [25]). The purpose of the present study was to investigate whether dispositional biases could be changed by exposing individuals to high/low spatial frequencies (Experiments 1ab and 2ab), or global/local forms (Experiment 3).

Through a series of 5 experiments, we measured dispositional global/local bias with a hierarchical forced-choice shape task (Experiments 1a, 2a, and 3), a traditional Navon letter task (Experiments 1b and 2b), and a high/low spatial frequency face task (Experiment 3). To manipulate global/local biases, we used high/low spatial frequency faces (Experiment 1), high/low spatial frequency gratings (Experiment 2), and Navon stimuli (Experiment 3). In 4 of 5 experiments we were unable to show significant differences in global bias following a manipulation. In general, the results suggest that dispositional global/local biases are stable across time, and resistant to recent exposure to high or low spatial frequency information.

Although we were unable to influence dispositional global/local biases in almost all of the attempts, we did find significant differences in global scores following the manipulation task in Experiment 2b. In Experiment 2b there was a significant difference in pre- to post-manipulation Navon global interference scores following the high spatial frequency grating manipulation (but not the low spatial frequency), and the interaction between pre/post and manipulation frequency approached significance for the Navon task. Although Experiment 2b provides rather weak evidence that Navon interference scores can be modulated by previous viewing of high/low spatial frequency gratings, if the finding is indeed real, why did the predicted pattern appear for only this one experiment? One consideration is that in this experiment we used the traditional Navon letter task as a dispositional measure. Previous research has shown that although the dispositional face and shape global/local tasks used here are remarkably stable over time (test-retest correlations of .70 or greater from two sessions held over one week apart), and are good individual differences measures, the Navon task is a much less reliable measure with test re-test correlations approximating .30 [45]. As such, it is possible that the Navon task is more open to transient state influences, and therefore was better able to capture pre-to-post manipulation changes in dispositional biases, whereas the forced choice face and shape tasks may be better measures of stable trait-like biases. Indeed, much of the work showing that global/local bias can be modulated has used Navon stimuli. For example, RTs on the Navon task can be altered by having participants perform simple tasks, such as estimating distances [24] or navigating obstacles in a maze [25]. Navon RTs can also be altered by inducing participants into an affective state that is high in approach motivation [3]. As such, the Navon task may capture more flexible global/local states, and this may partially explain why we were unable to show an effect of SF manipulation in most of our experiments. However, this pattern of results was not found for Experiment 1b, which used the same dispositional Navon measure. In addition, induced positive [4]and negative [5] affect has been shown to modulate scores on the hierarchical shape task used here, which shows that it is not just Navon performance that can be modulated. As such, it may be that there are generalized attentional processes that are unrelated to global/local per se, but that sometimes lead to small global/local-induced biases that are both difficulty to observe, and that are influenced by small experimental differences and nuances. As such, it is possible that the significant effect in Experiment 2b was found by chance and is not necessarily meaningful.

The primary purpose of the current experiment was to examine whether exposure to high and low spatial frequencies could alter dispositional global/local bias, thus 2 out of the 3 manipulation tasks used in this experiment were spatial frequency tasks. Spatial frequencies have been linked to global/local processing [49], but they are not necessarily global or local in and of themselves [54]. Indeed, Lamb and Yund [55] showed that the removal of low SFs can *slow* global processing, but does not eliminate global biases, nor does it affect the ability to switch attention from global to local forms. As such, although the face and grating manipulations have been used to influence performance on other tasks that have been linked to global/local processing (such as face identification), and high or low spatial frequency information in a given stimulus can facilitate global or local processing for that stimulus, the present results provide evidence that consistent exposure to information of high or low spatial frequency is not sufficient to alter subsequent global/local biases. This result may suggest that the mechanisms that underlie spatial frequency bias and global/local bias are independent, which supports the findings of Hills and Lewis [50].

Indeed, further evidence for the independence comes from the finding that manipulating participants with actual global/local stimuli in Experiment 3 did not alter their spatial frequency use in the hybrid face task. Furthermore, we found no effect on global/local bias in the hierarchical shape task when we exposed participants to global and local Navon letters in Experiment 3, suggesting that the processes underlying these two different global/local measures may also be independent. These suggestions are supported by a recent study showing that although the three dispositional measures used here (Navon letter interference, hierarchical shape choice, high/low SF face choice) are reliable over time, they are uncorrelated with each other, suggesting that they measure unique aspects of global/local processing [45]. Trying to manipulate global/local biases with a completely different type of task may not have been effective here because global/local biases that are measured by the hierarchical shape task may be immune to changes from the other tasks, as they are not tapping into the same aspect of global/local processing. As such, it might be more useful to examine whether dispositional biases can be manipulated by using the same stimuli for both the manipulation and to assess dispositional biases. Indeed, level-repetition effects in the literature show that individuals are faster to identify global/local information if they have recently completed trials that were presented at the same global/local level (e.g., faster RTs on global trials if the previous trial was also global; see [13]–[15]). Additionally, Lewis et al. [19] showed that face recognition was enhanced if the same type of Navon task (either globally- or locally-directed) was presented at both the learning and the retrieval face recognition stage, but impaired if there was a mismatch at encoding and retrieval. As such, dispositional biases on the Navon letter task might be more easily manipulated by presenting participants repeatedly with global or local Navon letters.

Ultimately, more research is needed to understand the exact nature of dispositional global/local biases, but these findings suggest that they are relatively immune to changes from exposure to high/low spatial frequencies and other global/local stimuli. A few studies have shown that attention to local or global levels of Navon stimuli can influence face recognition ability, where face recognition ability is better after attending to global levels than to local levels (e.g., [34], [35]). The present results suggest that such findings are unlikely to result from attention to global levels enhancing use of low spatial frequency information or from attention to local levels enhancing use of high spatial frequency information.

## Conclusions

In this paper we presented 5 different experiments that examined whether dispositional global/local biases could be altered by exposing participants to high/low spatial frequency information (Experiments 1ab and 2ab) or global/local Navon letters (Experiment 3). We used multiple measures of dispositional global/local bias (i.e., shapes, Navon letters, and faces,), and a variety of manipulation tasks (i.e., high/low spatial frequency faces, gratings, and Navon stimuli). Ultimately, we were unable to show the predicted pattern of significant changes from pre-to-post manipulation on the dispositional measures in 4 of 5 attempts, and showed significant weak for the other one. These findings provide evidence that global/local processing biases are relatively resistant to recent exposure to high or low spatial frequency information. Furthermore, consistent with previous results [45], [50], the present results provide further evidence for the independence of the mechanisms that underlie spatial frequency bias and global/local bias.

## References

[1] Navon D (1977) Forest Before Trees: The Precedence of Global Features in Visual Perception. Cogn Psychol 9: 353–383 10.1016/0010-0285(77)90012-3

[2] Navon D (1981) The forest revisited: More on global precedence. Psychol Res 43: 1–32 10.1007/BF00309635

[3] Gable P a, Harmon-Jones E (2008) Approach-motivated positive affect reduces breadth of attention. Psychol Sci 19: : 476–482.Available: http://www.ncbi.nlm.nih.gov/pubmed/18466409. Accessed 27 May 2013. 10.1111/j.1467-9280.2008.02112.x 18466409

[4] Fredrickson BL, Branigan C (2005) Positive emotions broaden the scope of attention and thought-action repertoires. Cogn Emot 19: : 313–332. Available: http://www.pubmedcentral.nih.gov/articlerender.fcgi?artid=3156609&tool=pmcentrez&rendertype=abstract. Accessed 22 May 2013. 10.1080/02699930441000238 PMC3156609 21852891

[5] Gasper K, Clore GL (2002) Attending to the big picture: mood and global versus local processing of visual information. Psychol Sci 13: : 34–40. Available: http://www.ncbi.nlm.nih.gov/pubmed/11892776. Accessed 27 May 2013. 10.1111/1467-9280.00406 11892776

[6] Kimchi R, Palmer SE (1982) Form and texture in hierarchically constructed patterns. J Exp Psychol Hum Percept Perform 8: : 521–535. Available: http://www.ncbi.nlm.nih.gov/pubmed/6214605. Accessed 27 May 2013. 10.1037//0096-1523.8.4.521 6214605

[7] Evans M a, Shedden JM, Hevenor SJ, Hahn MC (2000) The effect of variability of unattended information on global and local processing: evidence for lateralization at early stages of processing. Neuropsychologia 38: : 225–239. Available: http://www.ncbi.nlm.nih.gov/pubmed/10678690. Accessed 27 May 2013. 10.1016/s0028-3932(99)00080-9 10678690

[8] Fink GR, Marshall JC, Halligan PW, Frith CD, Frackowiak RS, et al. (1997) Hemispheric specialization for global and local processing: the effect of stimulus category. Proc Biol Sci 264: : 487–494. Available: http://www.pubmedcentral.nih.gov/articlerender.fcgi?artid=1688391&tool=pmcentrez&rendertype=abstract. Accessed 27 May 2013. 10.1098/rspb.1997.0070 PMC1688391 9149423

[9] Kinchla RA, Wolfe JM (1979) The order of visual processing: “Top-down,” “bottom-up,” or “middle-out.”. Percept Psychophys 25: 225–231. 461079 10.3758/bf03202991

[10] Kimchi R (1992) Primacy of wholisitic processing and global/local paradigm: A critical review. Psychol Bull 112: 24–38 10.1037/0033-2909.112.1.24 1529037

[11] Yovel G, Yovel I, Levy J (2001) Hemispheric asymmetries for global and local visual perception: Effects of stimulus and task factors. J Exp Psychol Hum Percept Perform 27: 1369–1385 10.1037/0096-1523.27.6.1369 11766931

[12] Paquet L, Merikle PM (1984) Global precedence: The effect of exposure duration. Can J Psychol Can Psychol 38: : 45–53. Available: http://doi.apa.org/getdoi.cfm?doi=10.1037/h0080783. Accessed 27 May 2013.

[13] Hübner R (2000) Attention shifting between global and local target levels: The persistence of level- repetition effects. Vis cogn 7: 465–484 10.1080/135062800394612

[14] Lamb MR, London B, Pond HM, Whitt KA (1998) Automatic and Controlled Processes in the Analysis of Hierarchical Structure. Psychol Sci 9: : 14–19. Available: http://pss.sagepub.com/lookup/doi/10.1111/1467-9280.00003. Accessed 27 May 2013.

[15] Lamb MR, Yund EW (1996) Spatial frequency and attention: effects of level-, target-, and location-repetition on the processing of global and local forms. Percept Psychophys 58: : 363–373. Available: http://www.ncbi.nlm.nih.gov/pubmed/8935897. Accessed 27 May 2013. 10.3758/bf03206812 8935897

[16] Robertson LC (1996) Attentional persistence for features of hierarchical patterns. J Exp Psychol Gen 125: : 227–249. Available: http://www.ncbi.nlm.nih.gov/pubmed/8751819. Accessed 27 May 2013. 10.1037//0096-3445.125.3.227 8751819

[17] Ward LM (1982) Determinants of attention to local and global features of visual forms. J Exp Psychol Hum Percept Perform 8: : 562–581. Available: http://www.ncbi.nlm.nih.gov/pubmed/6214608. Accessed 27 May 2013. 10.1037//0096-1523.8.4.562 6214608

[18] Shedden J, Marsman I, Paul M, Nelson A (2003) Attention switching between global and local elements: Distractor category and the level repetition effect. Vis cogn 10: : 433–470. Available: http://www.tandfonline.com/doi/abs/10.1080/13506280244000159. Accessed 27 May 2013.

[19] Lewis MB, Mills C, Hills PJ, Weston NJ (2009) Navon letters affect face learning and face retrieval. Exp Psychol 56: 258–264. 19439398 10.1027/1618-3169.56.4.258

[20] Fredrickson BL (2001) The role of positive emotions in positive psychology: The Broaden-and-Build theory of positive emotions. Am Psychol 56: 218–226 10.1037/0003-066X.56.3.218 11315248 PMC3122271

[21] Ashby F, Isen A (1999) A neuropsychological theory of positive affect and its influence on cognition. Psychol Rev 106: : 529–550. Available: http://psycnet.apa.org/?fa=main.doiLanding&doi=10.1037/0033-295X.106.3.529. Accessed 27 May 2013. 10.1037/0033-295x.106.3.529 10467897

[22] Gable P, Harmon-Jones E (2010) The blues broaden, but the nasty narrows: attentional consequences of negative affects low and high in motivational intensity. Psychol Sci 21: : 211–215. Available: http://www.ncbi.nlm.nih.gov/pubmed/20424047. Accessed 27 May 2013. 10.1177/0956797609359622 20424047

[23] Harmon-Jones E, Price TF, Gable P a. (2012) The Influence of Affective States on Cognitive Broadening/Narrowing: Considering the Importance of Motivational Intensity. Soc Personal Psychol Compass 6: : 314–327. Available: http://doi.wiley.com/10.1111/j.1751-9004.2012.00432.x. Accessed 27 May 2013.

[24] Liberman N, Förster J (2009) The effect of psychological distance on perceptual level of construal. Cogn Sci 33: : 1330–1341. Available: http://www.ncbi.nlm.nih.gov/pubmed/21585508. Accessed 27 May 2013. 10.1111/j.1551-6709.2009.01061.x 21585508

[25] Marguc J, Förster J, Van Kleef G a (2011) Stepping back to see the big picture: when obstacles elicit global processing. J Pers Soc Psychol 101: : 883–901. Available: http://www.ncbi.nlm.nih.gov/pubmed/21875228. Accessed 27 May 2013. 10.1037/a0025013 21875228

[26] Woltin K-A, Corneille O, Yzerbyt VY (2012) Improving communicative understanding: The benefits of global processing. J Exp Soc Psychol 48: : 1179–1182. Available: http://linkinghub.elsevier.com/retrieve/pii/S0022103112000522. Accessed 27 May 2013.

[27] Förster J (2009) Relations between perceptual and conceptual scope: how global versus local processing fits a focus on similarity versus dissimilarity. J Exp Psychol Gen 138: : 88–111. Available: http://www.ncbi.nlm.nih.gov/pubmed/19203171. Accessed 27 May 2013. 10.1037/a0014484 19203171

[28] Hanif A, Ferrey AE, Frischen A, Pozzobon K, Eastwood JD, et al. (2012) Manipulations of attention enhance self-regulation. Acta Psychol (Amst) 139: : 104–110. Available: http://www.ncbi.nlm.nih.gov/pubmed/22005394. Accessed 27 May 2013. 10.1016/j.actpsy.2011.09.010 22005394

[29] Large M-E, McMullen P a (2006) Hierarchical attention in discriminating objects at different levels of specificity. Percept Psychophys 68: : 845–860. Available: http://www.ncbi.nlm.nih.gov/pubmed/17076351. Accessed 27 May 2013. 10.3758/bf03193706 17076351

[30] Förster J, Dannenberg L (2010) GLOMO sys: Specifications of a Global Model on Processing Styles. Psychol Inq 21: : 257–269. Available: http://www.tandfonline.com/doi/abs/10.1080/1047840X.2010.507989. Accessed 27 May 2013.

[31] Tanaka JW, Farah MJ (1993) Parts and wholes in face recognition. Q J Exp Psychol A 46: : 225–245. Available: http://www.ncbi.nlm.nih.gov/pubmed/8316637. Accessed 27 May 2013. 10.1080/14640749308401045 8316637

[32] Valentine T (1988) Upside-down faces: A review of the effect of inversion upon face recognition. Br J Psychol 79: 471–491. 3061544 10.1111/j.2044-8295.1988.tb02747.x

[33] Hills PJ, Lewis MB (2008) European Journal of Cognitive Testing alternatives to Navon letters to induce a transfer-inappropriate processing shift in face recognition. Eur J Cogn Psychol 20: 561–576 10.1080/09541440701728524

[34] Macrae CN, Lewis HL (2002) Do I know you? Processing Orientation and Face Recognition. Psychol Sci 13: 194–196. 11934008 10.1111/1467-9280.00436

[35] Perfect TJ (2003) Local processing bias impairs lineup performance. Psychol Rep 93: 393–394. 14650660 10.2466/pr0.2003.93.2.393

[36] Weston NJ, Perfect TJ (2005) Effects of processing bias on the recognition of composite face halves. Psychon Bull Rev 12: : 1038–1042. Available: http://www.ncbi.nlm.nih.gov/pubmed/16615325. Accessed 27 May 2013. 10.3758/bf03206440 16615325

[37] Gao Z, Flevaris A V, Robertson LC, Bentin S (2011) Priming global and local processing of composite faces: revisiting the processing-bias effect on face perception. Atten Percept Psychophys 73: : 1477–1486. Available: http://www.pubmedcentral.nih.gov/articlerender.fcgi?artid=3118009&tool=pmcentrez&rendertype=abstract. Accessed 27 May 2013. 10.3758/s13414-011-0109-7 PMC3118009 21359683

[38] Davidoff J, Fonteneau E, Fagot J (2008) Local and global processing: observations from a remote culture. Cognition 108: : 702–709. Available: http://www.ncbi.nlm.nih.gov/pubmed/18662813. Accessed 27 May 2013. 10.1016/j.cognition.2008.06.004 18662813

[39] Stoesz BM, Jakobson LS, Kilgour AR, Lewycky ST (2007) Local processing advantage in musicians: Evidence from disembedding and contructional tasks. Music Percept 25: 153–165.

[40] Colzato LS, van Beest I, van den Wildenberg WPM, Scorolli C, Dorchin S, et al. (2010) God: Do I have your attention? Cognition 117: : 87–94. Available: http://www.ncbi.nlm.nih.gov/pubmed/20674890. Accessed 27 May 2013. 10.1016/j.cognition.2010.07.003 20674890

[41] Moritz S, Wendt M (2006) Processing of local and global visual features in obsessive-compulsive disorder. J Int Neuropsychol Soc 12: : 566–569. Available: http://www.ncbi.nlm.nih.gov/pubmed/16981609. Accessed 27 May 2013. 10.1017/s1355617706060577 16981609

[42] Scherf KS, Luna B, Kimchi R, Minshew N, Behrmann M (2008) Missing the big picture: impaired development of global shape processing in autism. Autism Res 1: : 114–129. Available: http://www.pubmedcentral.nih.gov/articlerender.fcgi?artid=2670479&tool=pmcentrez&rendertype=abstract. Accessed 23 May 2013. 10.1002/aur.17 PMC2670479 19360658

[43] Scherf KS, Behrmann M, Kimchi R, Luna B (2009) Emergence of global shape processing continues through adolescence. Child Dev 80: 162–177 10.1111/j.1467-8624.2008.01252.x.Emergence 19236399 PMC2648130

[44] McKone E, Aimola Davies A, Fernando D, Aalders R, Leung H, et al. (2010) Asia has the global advantage: Race and visual attention. Vision Res 50: : 1540–1549. Available: http://www.ncbi.nlm.nih.gov/pubmed/20488198. Accessed 27 May 2013. 10.1016/j.visres.2010.05.010 20488198

[45] Dale G, Arnell KM (2013) Investigating the stability of and relationships among global/local processing measures. Atten Percept Psychophys 75: : 394–406. Available: http://www.ncbi.nlm.nih.gov/pubmed/23354593. Accessed 23 May 2013. 10.3758/s13414-012-0416-7 23354593

[46] Deruelle C, Rondan C, Salle-Collemiche X, Bastard-Rosset D, Da Fonséca D (2008) Attention to low- and high-spatial frequencies in categorizing facial identities, emotions and gender in children with autism. Brain Cogn 66: : 115–123. Available: http://www.ncbi.nlm.nih.gov/pubmed/17693004. Accessed 25 May 2013. 10.1016/j.bandc.2007.06.001 17693004

[47] Dale G, Arnell KM (2010) Individual differences in dispositional focus of attention predict attentional blink magnitude. Atten Percept Psychophys 72: : 602–606. Available: http://app.psychonomic-journals.org/content/72/3/602.abstract. Accessed 27 May 2013. 10.3758/APP.72.3.602 20348566

[48] Shulman GL, Sullivan M a, Gish K, Sakoda WJ (1986) The role of spatial-frequency channels in the perception of local and global structure. Perception 15: : 259–273. Available: http://www.ncbi.nlm.nih.gov/pubmed/3797200. Accessed 27 May 2013. 10.1068/p150259 3797200

[49] Shulman GL, Wilson J (1987) Spatial frequency and selective attention to local and global information. Perception 16: : 89–101. Available: http://www.ncbi.nlm.nih.gov/pubmed/3671045. Accessed 27 May 2013. 10.1068/p160089 3671045

[50] Hills PJ, Lewis MB (2009) A spatial frequency account of the detriment that local processing of Navon letters has on face recognition. J Exp Psychol Hum Percept Perform 35: : 1427–1442. Available: http://www.ncbi.nlm.nih.gov/pubmed/19803647. Accessed 27 May 2013. 10.1037/a0015788 19803647

[51] Minear M, Park DC (2004) A lifespan database of adult facial stimuli. Behav Res Methods, Instruments, Comput Spec Issue Web-based Arch Norms, Stimuli, Data Part 2 36: : 630–633. Available: http://www.ncbi.nlm.nih.gov/pubmed/15641408. Accessed 27 May 2013. 10.3758/bf03206543 15641408

[52] Finger K (2002) Mazes and music: using perceptual processing to release verbal overshadowing. Appl Cogn Psychol 16: : 887–896. Available: http://doi.wiley.com/10.1002/acp.922. Accessed 27 May 2013.

[53] Mathôt S (2010) Online Gabor patch generator (software). Available: http://www.cogsci.nl/software/online-gabor-patch-generator. Accessed 27 May 2013.

[54] Sierra-Vázquez V, Serrano-Pedraza I, Luna D (2006) The effect of spatial-frequency filtering on the visual processing of global structure. Perception 35: : 1583–1609. Available: http://www.perceptionweb.com/abstract.cgi?id=p5364. Accessed 27 May 2013. 10.1068/p5364 17283927

[55] Lamb MR, Yund EW (1993) The role of spatial frequency in the processing of hierarchically organized stimuli. Percept Psychophys 54: 773–784. 8134247 10.3758/bf03211802

